# Method to Investigate Multi-Axis Release Action of Ski Safety Bindings: A New Approach for Testing in Research and Development

**DOI:** 10.3389/fspor.2021.585775

**Published:** 2021-02-10

**Authors:** Florian Nimmervoll, Roland Eckerstorfer, Johannes Braumann, Alexander Petutschnigg, Bruno Sternad

**Affiliations:** ^1^Industrial Design Department, Institute of Space and Design, University of Art and Design Linz, Linz, Austria; ^2^Department of Measurement Technology, Global Hydro Energy GmbH, Niederranna, Austria; ^3^Creative Robotics Lab, Institute of Space and Design, University of Art and Design Linz, Linz, Austria; ^4^Department of Forest Products Technology and Timber Construction, University of Applied Sciences Salzburg, Puch bei Hallein, Austria

**Keywords:** alpine skiing, binding release forces, product testing, force measurement, industrial robot, ski deflection, ski binding boot system, strain gauge

## Abstract

The authors developed and elaborated on a new method to release ski bindings by utilizing an industrial robot to simulate release movement showing a spatial repeatability of ± 0.06 mm. The parametric programming of the release parameters gave free control while executing repeatable release tests. A series of different motion patterns were performed, on the one hand, to test the applicability of the setup to the simulation of motion patterns and, on the other, to check for the impact of the ski deformations like ski deflections within the range of −5 mm to −85 mm, on the safety bindings' release forces. As certain falling mechanisms are related to knee injury, which is the most common severe injury in alpine skiing, this testing method can be used to develop related displacement movements in future. This movements do not necessarily accord with the directional release mechanics of safety ski bindings. The authors specify the developed testing apparatus as device for force measurements in 3D with an accuracy of ± 0.5% in boot-sole-plane. The intention behind this development is to enable faster, more versatile and adaptive testing procedures in R&D.

## 1. Introduction

Modern ski bindings were designed to protect the tibia and fibula from spiral and bending fractures and the accompanying standards such as ISO^1^ 9462, 8061, and 11088 are strongly related to this objective. Alpine ski bindings, the interface connecting the skier with the skis, have not fundamentally changed since the late 1970s (Natri et al., 1999). Basically, modern ski bindings possess a directional sideways release at the toe piece (front parts of the ski bindings) and an upwards release at the heel piece (back parts of the ski bindings). Since this type of safety ski bindings appeared in the early 1960s, traumata of the ankles and tibia fractures decreased substantially, whereas on the contrary knee injuries rates have not decreased substantially. Bearing in mind that research sources show (Finch and Kelsall, 1998; Langran and Sivasubramaniam, 2002; Pressman and Johnson, 2003a; Ettlinger et al., 2006; Pujol et al., 2007; Burtscher et al., 2008; Johnson et al., 2009; Ruedl et al., 2009; Ekeland and Rødven, 2012; Flørenes et al., 2012; Kim et al., 2012; Sabeti, 2013, Koehle et al., 2002)^2^, injuries of the skier's knee could not be substantially mitigated^3^. Furthermore, recent statistics (Greenwald and Toelcke, 1997; Ruedl, 2011; Schulz, 2013, 2016; Ruedl et al., 2014; Shea et al., 2014) demonstrate that knee injuries, especially ruptures of the anterior cruciate ligament *(ACL)* and medial collateral ligament *(MCL)*, are some of the most frequent injuries in alpine skiing with no signs of decline. As a matter of fact, the skier and subsequently the ski boot applies forces to the ski bindings in 3D space, whereas ski bindings react differently to release directions and loading positions. For example, during a backward-twisting fall, the forces apply beyond the boot's heel and the lever, which takes effect on the sideways release of the toe piece, is relatively long. Another example is the forward-twisting fall. When catching an edge in a forward twisting fall situation, the center of gravity of the skier moves forward along the force vector of the mass inertia. This can lead to false negatives^4^, because most heel pieces are solely built to release upwards.

Consequently, the most popular falling mechanisms related to knee injuries are as follows: *Slip-Catch, Landing Back-Weighted, Dynamic Snow Plow, Backward Twisting Fall*, and *Boot-Induced Anterior Drawer (BIAD*^5^*)*, which are found well-described in literature (Johnson et al., 1983, 1997; Aune et al., 1995; Koehle et al., 2002; Bere et al., 2011, 2013).

Within these falling and subsequent injury mechanisms concerning the human's knee structure, the valgus-external rotation mechanism and the phantom foot mechanism are the most common causes of structural overload (Ettlinger et al., 1995). Besides, a distribution change from backward twisting falls toward forward twisting falls has been observed (Ruedl et al., 2009) that could be explained by the introduction of shorter skis with narrower turn radii.

Ski bindings testing procedures such as those according to ISO 9462:2014 match with the basic mechanics of modern ski bindings, nonetheless late or no release happens when certain falling mechanisms come into play, despite a conforming SBB setup (Ski-Boot-Binding-System, ISO 11088-2018), which is recommended to be tested with mechanical testing devices by retailers and hiring outlets (Finch and Kelsall, 1998). Thus, the authors decided to develop a new testing setup including multi-axis displacement paths. These shall help to rapidly generate new testing load cases. In turn, this could lead to more elaborate product testing and R&D. For testing current and future binding models and concepts, the motivation was to find a new experimental method not to test a skiing set up according to ISO 9462:2014 or ASTM F504, but to simulate basic release patterns to determine if and how severe deviations in release forces are caused by constraining forces and deformations of ski and binding. Typically, ski binding test machines according to ISO 9462 standards apply combinations of forces and torques to the ski. To investigate forces occurring during multi-plane movements, the authors utilized parametric programming of a six-axis industrial robot to repeatably simulate complex movements of the ski boot's sole surrogate. ISO 11088-2018^6^ suggests independently of the tibia method^7^ or the skier's weight method, a legit setup range of 30% of binding release torques in the fall direction. This is due to the definition of ±15% steps in binding adjustment table and because of higher false negatives (51% compared to 32%; Ruedl et al., 2016) in binding release in females, lower bindings setups in females, and therefore a gender-dependant binding setup is already discussed by Posch et al. (2017b). To show the capabilities of this new method, several inclinations of the testing boot sole were added to the release movement paths. The testing of pure lateral release motion or lateral outwards-rotation of the heel, despite its importance, was avoided, because it would have led to repeated binding damage as there was no overload emergency stop implemented in the system. Nonetheless, the testing setup enabled the measurement of the forces in 3D occurring in the binding setup during simulating loads similar to those induced by back weighted landing, lateral forces by slip-catching an edge, high ski deflection with regard to the phantom foot mechanism and most of all a freely definable and movable release pivot point.

## 2. Materials and Methods

### 2.1. General Arrangement

**Figure 1:**
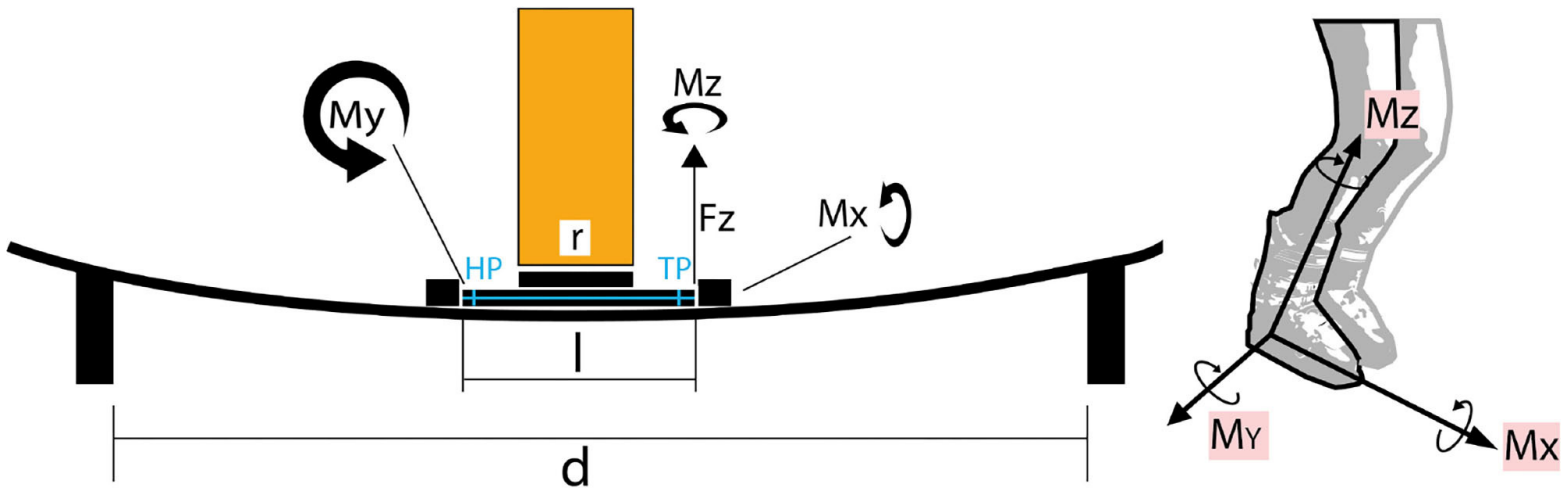
Definition of Cartesian coordinates and of the acting forces and torques, geometry of the test setup: length of ski boot replacing device *l*, length of connection flange *r*, distance between mounting support *d*, toe piece *TP*, heel port *HP*, force in positive z-direction *F*_*z*_, torques about x-, y-, and z-axis *M*_*x*_, *M*_*y*_, and *M*_*z*_, respectively.

**Figure 2:**
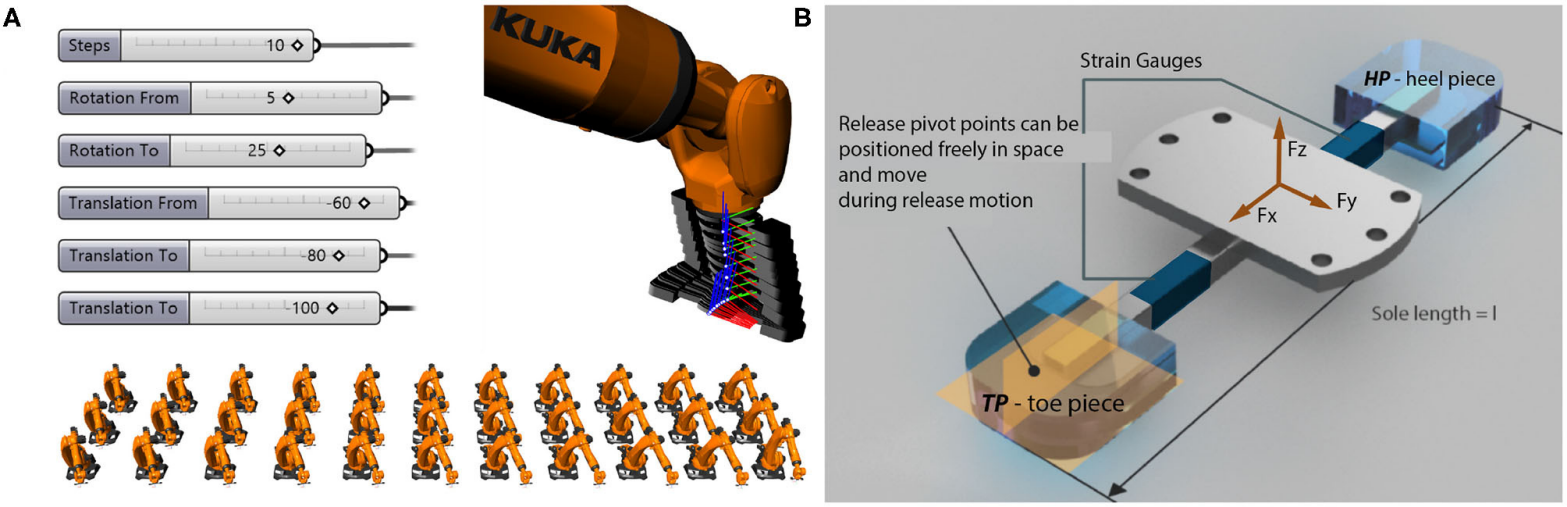
**(A)** Example of robot motion sequence and software user interface: Top: Image sequence of front piece release. Bottom: Inclined back-weighted toe piece release situation. **(B)** Ski boot replacing device made up of a connection flange in the middle welded to a steel-square-pipe, which is equipped with strain gauges located in the x-y-plane and the x-z-plane both toward the front and the back portion.

**Figure 3:**
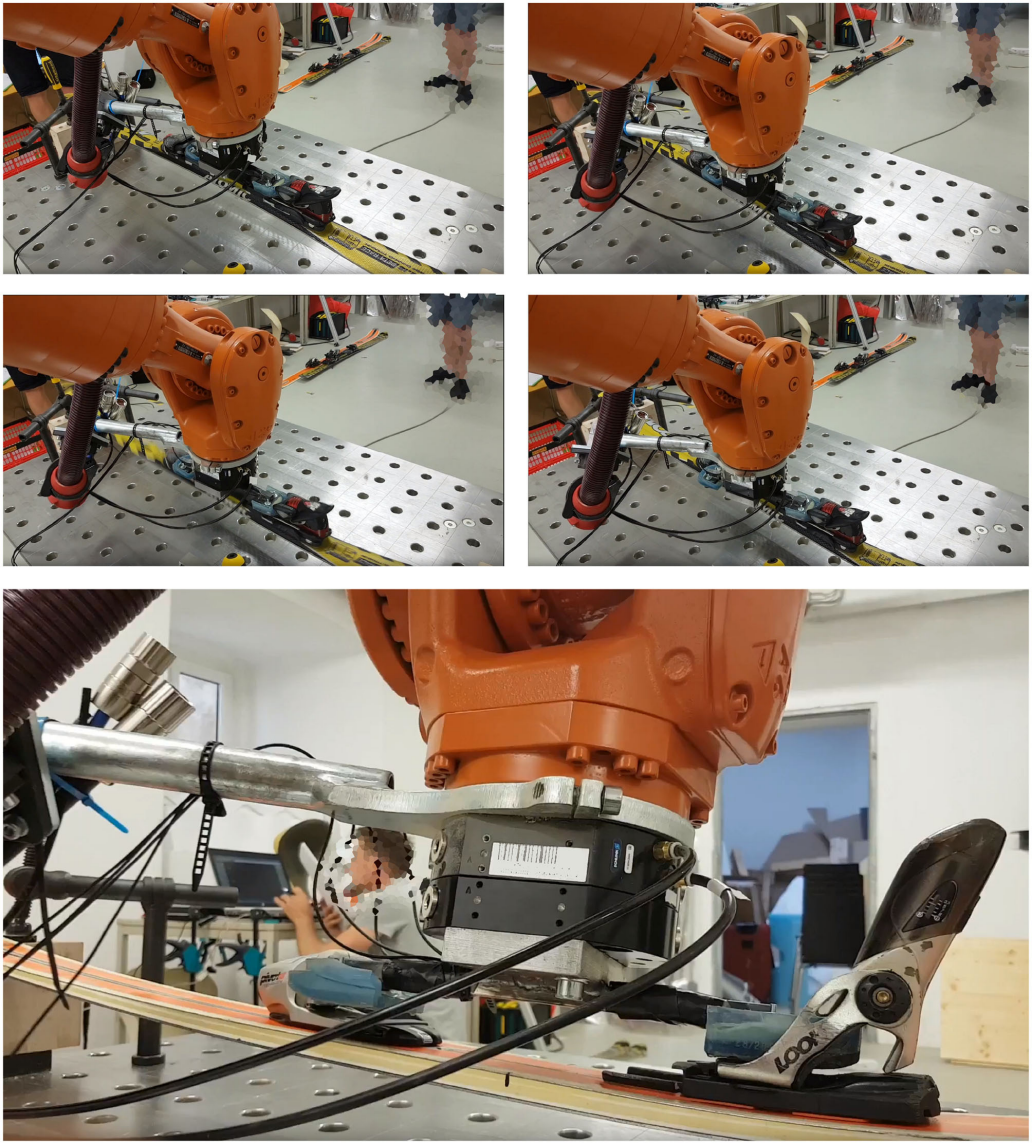
Photo sequence of an inclined back-weighted toe piece release situation: Top left: Ski in starting position (ski rests on supports without any inclination). Top right: Ski is deflected in negative z-direction. Mid left: Rotation around the y-axis to adumbrate skier's layback. Mid right: Toe piece release. Bottom: Toe piece release from zoomed side view.

Two types of skis and bindings^8^ were used for the tests. An end-effector equipped with strain gauges to measure the occurring forces during motion as well as the bindings' release forces was used instead of a standard ski boot to be placed in the binding. The end-effector was mounted on an industrial robotic arm (*KUKA Quantec series*), which performed the desired motion patterns (Figures 1, 2A, 3).

### 2.2. Ski's Arrangement

The ski was located on two supports, one in front and the other at the end of the ski. The distance between the supports was set at 130*cm*, while the bindings mounting center lines were positioned about 57*cm* from the back support (Figures 1, 2B). The ski ends were only fixated sideways for lateral release tests.

### 2.3. End-Effector

The end-effector consisted of a 15*mm* custom steel flange in the center to which two 80*mm* long steel-square-pipes having a wall thickness of 2*mm* were welded in opposite directions. The front and the back parts of this ski boot-replacing device was composed of an ISO 5355 alpine ski boot sole, which was form-fittingly attached to the steel structure using epoxy resin and additionally secured by four M4 screws each. Summing up, the arrangement was as follows, beginning from the back: heel piece (HP) from an alpine ski boot sole—steel-square-pipe with a wall thickness of 2–15mm thick and 100mm long steel flange—steel-square-pipe with a wall thickness of 2mm—toe piece (TP) of an alpine ski boot. The steel-square-pipes were both equipped with two strain gauge^9^ full bridges each located on half way between heel piece and flange, and flange and toe piece, respectively. On both sides, back and front, one full bridge is located in the x-y-plane in order to measure forces acting in the z-direction (indexed as *z*) and the second in the x-z-plane to determine forces in the y-direction (indexed as *y*). A general view of the end-effector design and setup is shown in Figure 2B. The steel-square-pipe with a wall thickness of 2mm was chosen because of its general easy availability, and its well-defined characteristics concerning deformation in combination with the applicability of standard general purpose strain gauges. The flange in the middle of the end-effector caused a mechanical separation of the front and back parts in terms of deformation, i.e., the forces in y- and z-direction, *F*_*y,TP*_ vs. *F*_*y,HP*_ and *F*_*z,TP*_ vs. *F*_*z,HP*_, could be detected independently from each other ^10^. So, during the tests, not only the bindings' release forces, *F*_*r*_, should be detected but also it should be reviewed if such a force measuring arrangement could be used for reverse engineering and motion pattern recognition, respectively. In other words, if it was possible to detect the pattern of the executed motion by just analyzing the measured forces. If motion pattern recognition turned out to be feasible and a similar measuring system could be applied to real ski boots, the force signals could be used to analyze the movements of the skier for the purpose of indicating looming falls and associated potential injuries. The full bridges were connected with an amplifier (*MGC Plus* by *HBM*) and an eight-channel data acquisition was used to record the output voltages of the amplifier (*DEWE43* data acquisition operating with the *Dewesoft 7.1* software by *DEWESoft*). A detailed calibration of the whole measuring chain was performed in the laboratory prior to the tests by means of using a primary standard in order to gain accurate coefficients for calculating the forces out of the measured voltage signals. Thus, *BEV*^11^-certified masses were attached to the end-effector for each full-bridge arrangement. The load was increased from small to high masses and decreased vice versa several times in order to eliminate imprecisions in the strain gauge application, inhomogeneities in load-induced deformations of the steel-square-pipes and to assure proper repeatability of the measurements. The obtained calibration coefficients turned out to result in an overall relative accuracy of ±0.5% over the total measurement range for all full bridges. Unfortunately, the full bridge of *F*_*y,HP*_ broke down due to bad attachment during the calibration. As this force measurement was of minor interest for the tests performed later and due to the lack of time for repairing until the accessibility to the industrial robot, it was not replaced by a new full bridge.

### 2.4. Industrial Robot

The end-effector was attached to an industrial robotic arm via the steel flange. The robotic arm (*KUKA Quantec series*), rated for a payload of 210 kg and a repeatability of ±0.06 mm according to ISO 9283, produced the release motions using displacement control. Industrial robots enabled a focus on the test setup itself to simulate the desired release motions by design, rather than requiring the construction of a bespoke, expensive testing apparatus. The visual programming environment *Grasshopper* was utilized, where parametric tool paths can be defined by connecting nodes that expose geometric and mathematical functions. Relevant variables such as the heel rotation and force could be exposed as number sliders, enabling rapid iterations of different load cases, not just in preparation but also on-site during testing. The robot simulation software (*KUKA|prc*) was directly linked to the parametric tool paths within *Grasshopper* so that they could be immediately evaluated, thus enabling an interactive feedback loop. The tool paths were then translated into *KUKA Robot Language* and sent to the robot controller. Using those tools, nearly 200 testing configurations were generated, with further fine-tuning happening on-site (Figures 2A, 3).

### 2.5. Testing

Basically, the tests should give evidence on the applicability, accuracy and ability to gain detailed information about force distribution in the sole-plane during release action of the newly developed method. So, it was not the aim to develop just a new method to test the functionality of safety bindings according to ISO 9462 but to simulate basic release patterns to determine if and how severe deviations in release forces are caused by constraining forces and deformations of ski and binding. Furthermore, the design of the end-effector and its ability for reverse engineering concerning motion pattern recognition should be tested. As the measurement setup applied to the end-effector presented in this paper was kept rather simple, the motion patterns performed by the robot were kept straightforward, too. As a result, the executed motion patterns neither meet the requirements of ISO standard nor correspond to complex falling trajectories. The motion patterns give an outlook of the capabilities of boot-sole-plane force measurements.

Generally, test sequences on the safety binding's back part, hereinafter referred to as *heel piece opening*, and on its front part, referred to as *toe piece opening*, were performed.

The heel piece opening sequence comprised a rotation around the y-axis, adumbrating the forward lean out-of-balance of a skier, and different deflections in z-direction. The center of rotation was decided to be located at the tip of the boot, i.e., at the front part of the end-effector, for two reasons: first, center of rotations located closer to the heel would cause further bending beyond the preset deflection in z-direction. Besides, further deflections may have caused severe damage of the skis. Second, the center of rotation's location anywhere else but in the shoe tip would result in more complex deformation shapes of the ski than just simple bending, like kinds of s-shaped deformation. The influence of more complex contortion profiles should be avoided as only the influence of different z-deflections on resulting release forces was of interest during the tests. The authors tested release movement with 5mm (flat ski), 30mm, and 60cm z-deflection to see the effect of compressive stress acting on the boot due to ski bending. The displacement in z-direction was measured below the center of the ski boot replacing device from the bottom of the ski surface downwards. A value of *z*=0mm meant that the ski was lying freely without any force being applied to it. The deflection values for the testing were chosen according to a related testing procedure of Supej and Senner (2017), who distinguished between three ski-deflection conditions. A flat ski position, a ski deflected according to ISO 9462:2014 referencing a 150cm support distance with 6cm deflection, and also a ski-deflection of 6cm but exceeding the ISO condition by reducing the support distance to 110cm. In the actual setup, the support distance of 130cm seemed to be an appropriate fit for both tested ski length (setup A 156 cm/setup B 186 cm). Finally, an additional rotation around the x-axis was applied to the 30mm z-deflection before the release motion was executed. The heel piece opening tests were only performed with setup A (*Atomic*). For comparison, Ahlbäumer et al. only moved the ski tip 10mm out of the longitudinal axis to simulate the rotational component while falling without applying any deflection to the ski nor changing the release force direction (Ahlbäumer et al., 1999).

During the toe piece opening test series, falling sequences like BIAD^12^, valgus external rotation and Phantom foot mechanism were intended to be allusively be simulated with four different ski deflections. The z-deflections were equal to those described in the heel piece opening sequences (*z* = −5mm, *z* = −30mm, *z* = −60mm) and additionally an extreme ski bending of *z* = −85mm was examined. For reference, Yoneyama et al. measured up to −30mm deflection in the ski's rear part while skiing 28m turns at about 70km/h (19.44m/s) (Yoneyama et al., 2010). A rotation of 5° around the y-axis with its center of rotation located in the middle of the front and the back part of the binding should indicate a dislocation of a skiers center of mass to the back. The release motion was a lateral movement of the end-effector's front piece, i.e., a rotation around the z-axis with its center of rotation in the heel part in order to find the release force of the safety binding's toe piece solely. If the center of rotation had been chosen elsewhere, an additional lateral force would have been induced in the binding's back part, too, and a more complex (but more realistic load distribution concerning real falling situations) would have resulted. The test sequences were done with the setup B (*Dynastar-setup*). Next, the influence of an additionally applied rotation around the x-axis of 10° at a z-deflection of −60mm and y-rotation of 5° on the release forces was checked for both ski setups, A (*Atomic*) and B (*Dynastar*). The toe piece opening test series required an extra lateral fixation of the ski to its supports to preclude the ski from slipping sideways on the supports.

Each test was repeated at least five times and a Student's *t*-test was applied assuming normally distributed measurement samples. The final data analysis was done in post-processing scripts (*Python 3.6.5*). The mean value and its 95% confidence level of the repetitions was calculated to check for significant differences in release forces due to the different ski deflection parameters.

## 3. Results

### 3.1. Motion Pattern Recognition

#### 3.1.1. Heel Piece Openings/Forward Bending Release

**Figure 4:**
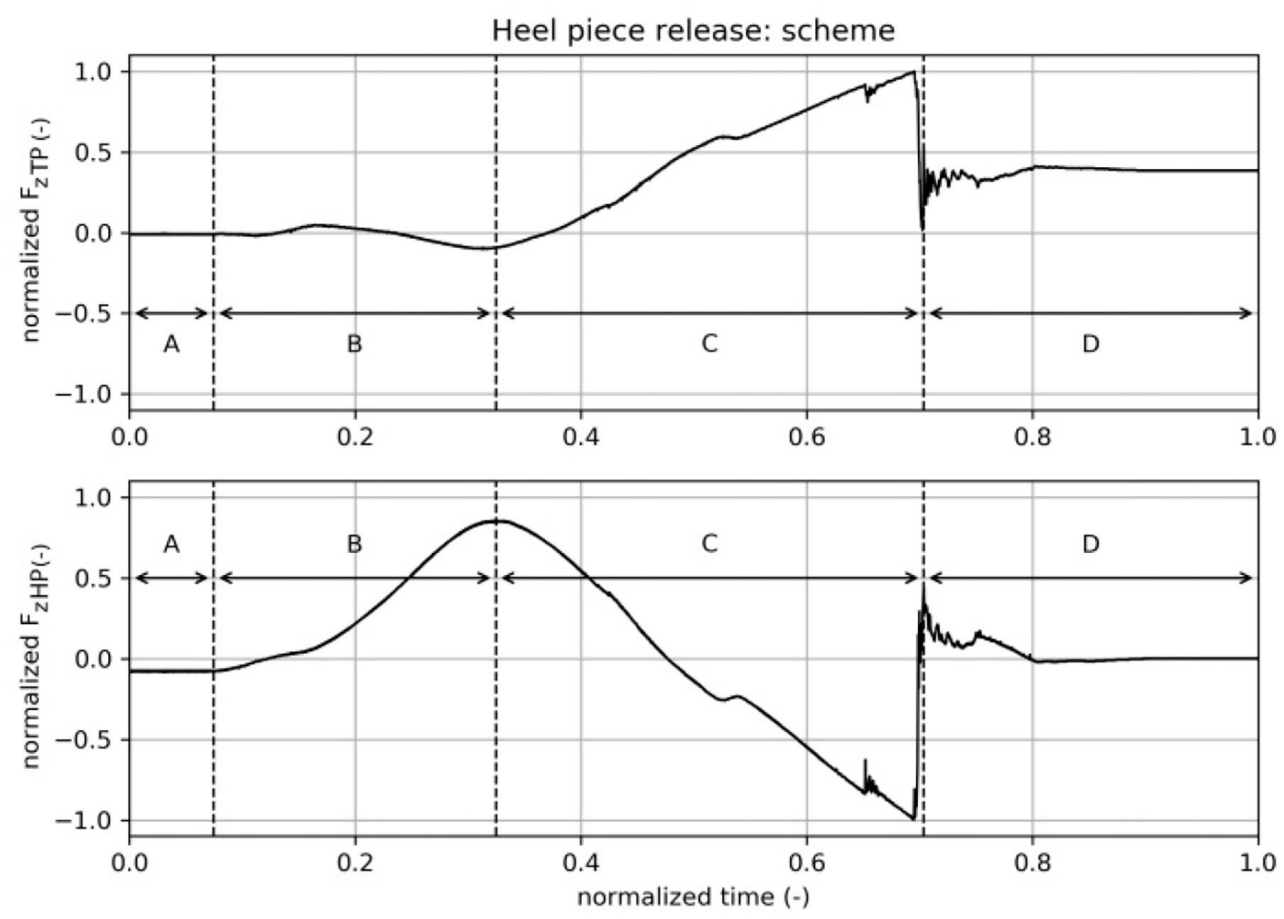
Heel piece release motion scheme, time signals: A—boot rests in binding, B—deflection of ski, C—boot heel moves up, D—boot released.

An example of measured time signals of the acting forces during the test of a heel piece release motion scheme (*z* = −5mm, no additional rotation around the x-axis) is depicted in Figure 4. During this test, no lateral force, i.e., in the y-direction (*F*_*y*_ = *const*. = 0), was applied, so only the recorded forces in z-direction, *F*_*z,TP*_ and *F*_*z,HP*_, are shown in this figure. The analysis of the time signals emphasized the strength or rather the novelty of the developed measuring setup as the movement pattern could be identified by the interpretation of the forces. The signals presented in Figure 4 were normalized to the maximum force that appeared during this test because absolute values were of minor interest and would only distract the reader from the focus on the method's capability of reverse engineering. Especially, the forces due to z-deflection could be misinterpreted as representing a skier's mass, but these forces are only related to the stresses and strains in the ski caused by its deformation. The time was also normalized, because the tests were also conducted at different speeds and, interestingly, it turned out that for all tests described in this paper, motion speed does not affect the results. For the purpose of easier interpretation and latter explanations of the time signals, Figure 4 is divided into four sections, denoted as A to D.

*A – boot rests in binding:* *F*_*z,TP*_ = 0*N*, whereas *F*_*z,HP*_ ≠ 0*N*, which was caused by sub-optimal positioning of the boot, i.e., the boot's and the binding's surfaces at the heel portion were not perfectly aligned in parallel in the x-y plane resulting in a slight rotation around the y-axis of less than 1°. However, the heel piece's release value was not affected by this.

*B – deflection of the ski in negative z-direction:* In the time signal of *F*_*z,HP*_, the ski's deflection force can be seen. The higher the peak at the end of section B, the bigger the deflection, the bigger the load applied to the ski. *F*_*z,TP*_ < 0*N*, due to the mechanical properties of the ski. The length from the mounting at the back part of the ski to the heel piece was much shorter than the length from the front portion to the front mounting of the ski, which caused a kind of horizontal s-shaped deformation resulting in a negative force acting at the front portion of the ski boot replacing device.

*C – boot heel moves up, with boot tip as center of rotation:* The minimum value in *F*_*z,HP*_ at the end of this section gave the release value of the binding heel piece. *F*_*z,TP*_ increased to even higher positive values within this section, which was related to an imperfect center of rotation at the front end of the front portion close to the anti-friction device. The bigger the displacement in z-direction, the higher the maximum *F*_*z,TP*_ value. However, the heel piece's release values were not influenced by alternating *F*_*z,TP*_ forces.

*D – boot released:* In this section, the boot's heel portion was released from the binding's heel piece, resulting in a final value of *F*_*z,HP*_ = 0*N*, whereas the front of the boot still remained in the binding. Thus, *F*_*z,TP*_ ≠ 0*N*, showed a final non-zero value.

#### 3.1.2. Toe Piece Openings/Backward Lateral Release

**Figure 5:**
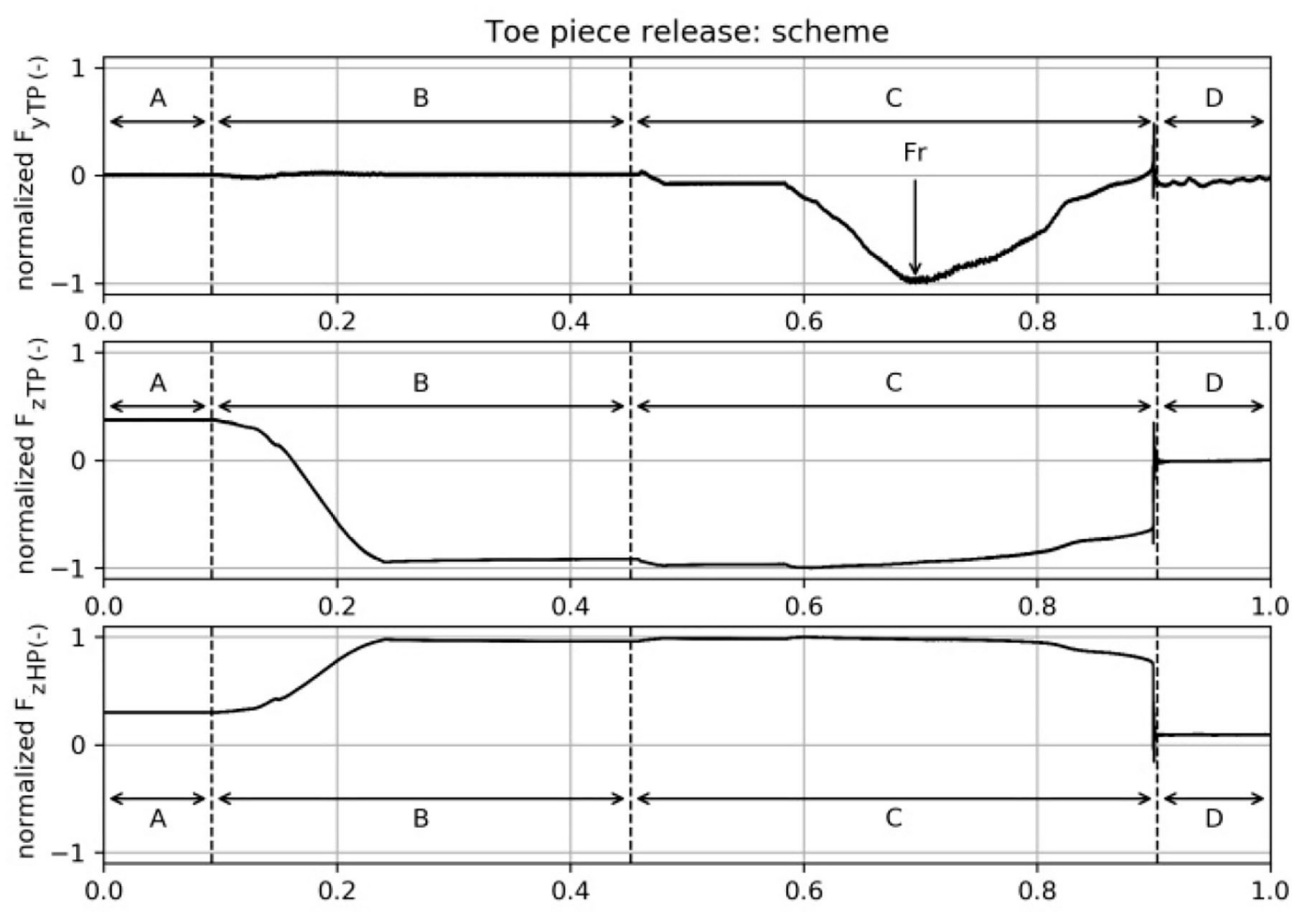
Toe piece release motion scheme, time signals: A—boot rests in binding, B—center of mass moves backwards, C—lateral movement of the boot tip, D—boot released (*F*_*r*_. release force).

Time signals of a test of the toe piece opening motion scheme are presented in Figure 5 for the purpose of demonstrating the ability to identify the movement pattern by analyzing the time signals. As previously described, there is an inclination in z-direction, followed by rotation around the y-axis of 5 with the center of rotation (*COR*) located in the middle of the boot replacing device. The heel portion was even more deflected in negative z-direction, whereas the front portion moved back up a bit. The rotation around the y-axis should simulate a back-weighted falling situation. Finally, the lateral release of the binding's front piece was performed by rotating the ski boot replacing device around the z-axis with the center of rotation being placed at the heel end. A normalization of the forces and time was done for the same reasons already mentioned in the description of heel piece openings. The plot is divided into four sections to make explanations easier.

*A – boot rests in binding, ski already deflected in negative z-direction:* Both forces in z-direction are positive (*F*_*z,TP*_ > 0 *N, F*_*z,HP*_ > 0 *N*) due to deflection in negative z-direction as the bending of the ski causes strains within the ski structure, which act against the end-effector.

*B – center of mass moves back:* In this section, a movement of the center of mass to the back of a skier was imitated by a rotation around the y-axis resulting in a decrease of *F*_*z,TP*_(*t*_*B*_) < 0 *N* < *F*_*z,TP*_(*t*_*A*_) and an increase of *F*_*z,HP*_(*t*_*B*_) > *F*_*z,HP*_(*t*_*A*_) > 0 *N*, respectively. The center of rotation was located in the middle of the boot sole. In the scheme depicted in Figure 5, the robot rested in this position for a period of time before section *C* was initiated.

*C – lateral movement of the boot tip, center of rotation at back of heel:* Within this period, the boot performed the lateral movement of the boot's tip in order to get the corresponding release force *F*_*y,TP*_ = *F*_*r*_, where *F*_*r*_ = min(*F*_*y,TP*_). The center of rotation was located in the back end of the heel and the boot rotated around the z-axis. It was observed that there was no sudden stepwise change of *F*_*y,TP*_ when it exceeded the release force *F*_*r*_ but increased steadily to *F*_*y,TP*_ = 0*N*. In the first part of section *C*, the measured force *F*_*y,TP*_ decreased to its minimum value and the lever arm of the ski binding's front part generated a counteracting force to the lateral movement, i.e., mainly in positive y-direction. At the position where *F*_*y,TP*_ = *F*_*r*_, the highest part of the boot front plate's curvature was reached and the binding's lever arm induced mainly a force in negative x-direction, i.e., the binding clamped the boot from toe to heel in the y-z plane instead of the initially induced clamping of solely the toe piece in the x-z plane. Later, the boot's curvature decreased, i.e., the center line of the boot has passed the tip of the binding's lever arm, resulting in a decrease of the clamping force until the toe was fully released. The effect of clamping the boot in the y-z plane induced changes of the forces *F*_*z,TP*_ and *F*_*z,HP*_, especially in the latter part of section *C*.

*D – boot released:* The boot's toe piece was fully released. As a result, the forces *F*_*y,TP*_ = 0*N* and *F*_*z,TP*_ = 0*N* as the toe piece did not touch any part of the binding any longer, whereas the heel was still located in the binding's back part, thus *F*_*z,HP*_ > 0 *N*. *F*_*z,HP*_(*t*_*D*_) < *F*_*z,HP*_(*t*_*A*_) due to the decreased internal forces, i.e., stress and strain, in the ski as a consequence of the ski's change in deflection shape.

### 3.2. Release Forces

**Figure 6:**
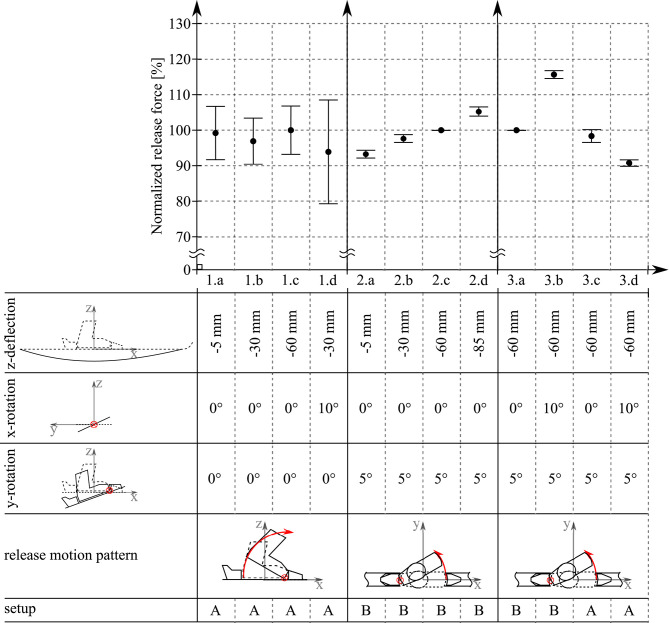
Release forces and 95% level of confidence for different release motion patterns.

For the determination of the release forces, each motion pattern was repeated at least five times in order to allow a proper statistical evaluation taking a Student's *t*-test into account assuming a normal distribution of the collected data.The mean value and its corresponding 95% level of confidence was calculated and displayed in Figure 6 as circles for the mean values and error bars representing the 95% confidence level. If the collected data of one deflection group lay outside the 95% confidence level range of another deflection group, a significant difference in the two groups can be assumed, i.e., the deformation of the ski does significantly influence the release force of the heel piece opening mechanism.

#### 3.2.1. Heel Piece Openings/Forward Bending Release

The measured release forces and confidence levels were normalized using the absolute values of the *z* = −60*mm* deflection scheme (Figure 6, *1.c*). As the tested safety bindings were of different age and design (Atomic binding contained a robust binding plate which the Look binding did not have), the examined ski's were of different lengths, as well as the release mechanism's adjustment may not be absolutely identical, it was decided to normalize, because the authors were not interested in the quality or rating of individual brands. The *z* = −60mm test was chosen for normalization as this deflection depth was also evaluated during all other test series. The measurement results did not show any statistically significant influence of the motion pattern concerning the heel piece release forces. Thus, a sole alternating horizontal bending of the ski did not affect the release force of the heel piece opening mechanism. However, taking a closer look at the single confidence levels, it could be seen that the additionally applied rotation of 10° around the x-axis caused a doubling in the release force's variance (Figure 6, *1.d*: approximately ±15%) compared to the previous tests without x-axis-rotation (Figure 6, *1.a* to *1.c*: approximately ±7%). The higher variance in release forces could indicate that the safety binding's release mechanism is affected by such a ski deformation, which results in a less accurate triggering of the opening apparatus.

#### 3.2.2. Toe Piece Openings/Backward Lateral Release

The measured release forces and confidence levels were normalized using the absolute values of the *z* = −60*mm* deflection scheme (Figure 6, *2.c*). Motion pattern *2.c* was repeated seven times but unfortunately, the first five tests turned out to be erroneous as closer inspection of the time signals revealed different behavior of these tests compared to the rest of the whole test series. A detailed analysis of the photo and video documentation confirmed that during these first five tests a systematic error in the mounting of the setup occurred. As a result, there were only two valid samples of pattern *2.c* available, which did not allow a proper statistical analysis other than evaluating its mean value. However, the whole test series showed an increase in the release forces with increasing deflection of the ski in negative z-direction. Apart from pattern *2.c*, the test results differ statistically significantly from each other. As the release forces seemed to increase linearly with increasing deflection, an extreme deformation with *z* =−85mm was also investigated. It turned out that even such an unnatural bending of the ski still allowed for assuming a linear relationship.

Finally, the influence of an additionally applied 10° rotation around the x-axis on the toe piece release force at a deflection of *z* = −60*mm* was observed for both ski and safety binding setups. A comparison of the 0° vs. 10° x-axis rotation for ski setup *B* (Dynastar setup) and *A* (Atomic setup) is shown in Figure 6, *3.a* vs. *3.b* and *3.c* vs. *3.d*, respectively. All the presented values were normalized by pattern *3.a*^13^. It could be seen that the two binding setups did not differ significantly for the tests without additional x-rotation (Figure 6, *3.a* and *3.c*). The small deviation in their mean values could be due to slightly different adjustments of the bindings' safety mechanisms. The application of a rotation around the x-axis resulted in significantly different release forces for both ski setups. Interestingly, the rotation caused a significant force increase in setup *B*, whereas in setup *A* the release force decreased.

## 4. Discussion

### 4.1. Release Situations

*Heel piece release:* As shown above, applied forces for heel piece release along the sagittal plane did not show any significant aberration of release forces, whereas inclining the test ski on x-axis during deflection loading lead to a greater dispersion. *Toe piece release*: The results demonstrated that BIAD falling mechanisms should be achievable to simulate with this method in future. More elaborate binding types^14^ could be tested more severely without breakage of the ski-boot-binding system, which could reveal clearer aberration in release forces in the Cartesian x-y-z directions relative to the ski inclination. This is of high interest because ACL strains correlate with the movement of the body's center of gravity and the movement of the skis connected to the snow surface. The resulting quadriceps loading produces anterior tibial translation^15^ and intrinsic strain to the ACL (Demorat et al., 2004). In an inclined back-weighted toe piece release situation (Figure 3), constraining forces were observed by an altered distance between sole and toe piece gliding plate. Additionally, upward forces on the toe piece release levers varied greatly. In skiing, this situation could lead to delayed or no binding release resulting in injury or binding damage.

### 4.2. Advantages, Limits, and Constraints

The tests demonstrated that quasi-static binding testing for research purposes works precisely and release displacement can be simulated and programmed fast. Pivot points were measured manually and aligned with the robot coordinate space. Hereby, a normalized procedure to define pivots would strengthen the informative value of this method. This bench test allowed for accurate testing and prompt adjustment of movement paths. As this apparatus had a fixated ski boot sole, the synchronized movement on different spatial planes was possible. Although the robot was very accurate with a spatial displacement accuracy of ±0.06mm and the end-effector was calibrated by means of a primary standard resulting in a relative uncertainty of ±0.5% over the total measuring range, it could not fully be guaranteed that any systematic errors occurred in the test setup. In order to further suppress systematic errors and thus increase the overall accuracy of the testing setup, an *in situ* calibration would be required. Unfortunately, such an *in situ* calibration apparatus would be rather complicated to be designed and realized.

Regarding the aforementioned experiments, execution speed of the release did not shown any significant aberration. Different acceleration patterns in loading were investigated. Key factors with reference to release forces definitely were as follows:

1. Ski bending and counter pressure.
2. Type of supports especially when executing twisted release motion.

A defined negative form to bend the ski against would additionally help to simulate boot induced back weighted fall mechanisms.

Tests with one support on either the front or the back side of the ski would result in more realistic representations of load distributions during falling schemes, i.e., during back weighted landing, respectively, BIAD situations the back part of the ski is strongly bent against the back support, whereas the front part would move freely, and vice versa for slip-catch and dynamic snow plow injury mechanisms. Nonetheless, the supports only fixated the ski sideways to avoid any support-induced ski bending. Especially for testing more extreme displacement paths, a stop signal should be implemented when an overload is measured to avoid equipment damage. This would also apply for high-dynamic testing because of its hard predictability. The developed measurement setup implemented in the ski boot replacing device showed that it was possible to ascertain the motion pattern applied to the device by interpreting the time signals. Transferring such a measurement setup to a real ski boot in combination with a mobile real-time analyzing tool could lead to the development of new safety bindings, which adjust the release mechanisms based on the motion pattern recognition. However, the method presented in this paper was only tested in the artificial surrounding of a laboratory using rather simple movements performed by an industrial robot. Furthermore, as the measurement setup applied to the end-effector was kept rather simple for the tests presented in this paper, the motion patterns performed by the robot also were kept simple. It is obvious that the presented setup cannot both be easily applied to ski boots and detect rather complex falling situations during real skiing, but it could be used as a kind of starting point for further more sophisticated developments. To develop knee-specific three-dimensional release testing paths that are more representative, in consideration of knee injuries and its counterpart, the pre-release malfunction of a ski safety binding, and motion capturing of artificial falling situations, respectively, musculoskeletal analysis computer models would enable further insight for expert evaluation.

This study was conducted to evaluate the accuracy and the constraints of this new method utilizing boot-sole-plane measurements. Basic release paths as well as twisted release movements with the focus on the backward loading of the bindings were put in the foreground of the described testing. As a result, several multiaxial release actions were calculated to compare aberrations in force loading patterns. Different release speeds did not show significant deviations in release forces over the conducted tests. Release speed was not included into further analysis here. The method allows quick changes of the displacement path triggering the release action of the binding. The high precision movement of an industrial robot and the iterative control over the movements through parametric programming are beneficial aspects of this setup. This might be of interest for the future work within following applied research topics.

## 5. Practical Applications

### 5.1. Release and Retention Behavior Testing of Ski Bindings

Supej et al. (Supej and Senner, 2017), for instance, have shown elaborately that modern bindings, no matter if mounted directly or mounted on a binding plate, were within the generous boundaries of DIN ISO 11088. In particular, there is an ongoing discussion^16^ about binding settings for female skiers, who could profit from lowered release settings within the norm^17^ (Laporte et al., 2009; Posch et al., 2017a,b) and failure of binding release seems to be about 20% higher for females compared to males (Ruedl et al., 2015, 2016). There is also a significant higher amount of false negatives during backward falling situations compared to forward falling (87 vs. 72%, *p* = 0.002) (Ruedl et al., 2015). But it is a tightrope walk between the reliable release of mechanical ski bindings in twisting fall situations^18^ such as described in Pressman and Johnson (2003b), Bere et al. (2013), Bere et al. (2011) and the danger of pre-release malfunction (false positives). The further development of representative release paths via 3D motion tracking could help to emulate the release action of forward twisting falls and backward twisting falls with the robot.

### 5.2. New Product Development: Thinking Out of the Box

Beginning in the 1980s, Hull et al. (Hull and Allen, 1981, Eseltine and Hull, 1991) made first approaches on developing electromechanical ski bindings that can handle a more sophisticated differentiation, which triggers the binding release action (see Gulick and Mote, 2001). Especially research groups around Senner (Senner et al., 2013; Senner et al., 2014; Nusser et al., 2016) regularly brought electromechanical approaches into discussion on the search for a way to reduce knee injuries. Recent mechanical binding models address knee injuries by adding degrees of freedom to the toe piece. Ahlbäumer et al. (1999) investigated the possibilities and the restrictions of toe-bindings with multi-directional release while applying backward fall related forces using testing devices for ski bindings according to ISO 9462 IAS 100. Concerning this matter, it is still unclear how vertical release forces should correlate with the lateral release settings of the toe piece and how false positives could be avoided. Similarly, some bindings^19^ offer a lateral release option in the heel piece of the binding.

This method can be useful in future to test new electronical or mechanical release features more distinctively. It can also be used for stress analysis of new components or the analysis of material behavior (Knye et al., 2016).

In developing new skis, S-B-B^20^ combinations, the detailed knowledge of bending profiles and ski behavior is key information. This method can easily be applied for those demands and even simulate the dynamic loading of the S-B-B system. The robots' limits in acceleration speed and deceleration speed while applying critical forces to the S-B-B system allow high dynamic testing^21^ of modern S-B-B setups. For example, by integrating the shake behavior of skiing at high speeds as a test parameter. Nonetheless, the testing of new binding developments in view of slow speed falls seems to be of great importance, because of the relation between release speeds and the timing of muscle contractions (Aune et al., 1995). The applied force loads and vibration of the robot could be aligned with empiric on-piste data from other studies (Gilgien et al., 2013, 2015; Fasel et al., 2016; Spörri et al., 2017). The addition of resilient negative bending profiles (ski slope surface) to the setup will also enable the integration of ground reaction forces and should be considered for further development (Müller, 1994; Babiel et al., 1997; Nakazato et al., 2011). Furthermore, the integration of inverse kinematics in the programming would allow the calculation of position and rotation data of a virtual knee.

The plausibility to integrate an artificial lower leg and knee in the system needs to be assessed by bio-mechanical experts. Knee surrogates, for example, have been used to evaluate knee braces used especially in contact sports^22^ (Cawley et al., 1989; Brown et al., 1990) and lately the evaluation of knee braces and ski safety bindings in alpine skiing (Nusser et al., 2016).

Furthermore, in the production lines of high-quality ski models, manual testing and bending of skis is an important part of the final fine tuning. To that effect, this method has to be tested and developed further to gain more insight into how those processes could be assisted or replaced.

## 6. Outlook

While there are plenty of solutions to mechanically release a binding in multiple directions, the authors assume it could be revealing, if force loading patterns can be diagnosed somewhere in the skiing equipment, that can be directly related to knee injury related falls. This means only specific overloading leads to an early release of an “intelligent” ski binding. We assume the integration of a sensor-equipped ski boot (Nimmervoll et al., 2020) as well as the integration of sensors that are positioned in the ski bindings' interfaces (Nakazato et al., 2011; Martínez Álvarez et al., 2020) as promising options for future testing. Force data from the slopes can be integrated in data analysis that allows more detailed studies of interrelations between binding forces and measured forces in the ski boot. To gain more insight into the assumption that occurring forces can be reliably differentiated between, one could say, sportive skiing load patterns and ACL endangering load patterns, the authors searched for versatile method to measure forces in laboratory conditions. Referring to this, this experiment is a promising endeavor to correlate prospective real-world data with robot simulation data in future studies.

## Data Availability Statement

The raw data supporting the conclusions of this article will be made available by the authors, without undue reservation.

## Author Contributions

All authors contributed to the study conception and the design.

## Conflict of Interest

RE was employed by the company Global Hydro Energy GmbH. The remaining authors declare that the research was conducted in the absence of any commercial or financial relationships that could be construed as a potential conflict of interest.

## Footnotes

^1^ISO-International Organization for Standardization.

^2^Especially Koehle et al. (2002) address the ski binding design as one of the main preventative factors.

^3^Flørenes et al. (2012) have clearly shown the statistical importance of knee injuries, especially during snowboarding, freestyle skiing, and alpine skiing. In addition, Bere et al. (2011) illustrate correlations of several knee injury mechanisms concerning professional athletes and recreational skiers.

^4^Failure of binding release.

^5^The boot induced mechanism occurs when a skier lands off balance to the rear while attempting a jump. Instinctively, the skiers leg fully extends. As a result, the skier lands on the tails of the ski that forces the back of the ski boot against the calf. This pushes the tibia forwards relative to the femur and the ACL tears as a result. A similar mechanism occurs when a stationary skier is hit from behind on the lower leg (usually by another skier or snowboarder). This again applies sudden pressure on the back of the calf, forcing the tibia forwards in a similar manner to that described above with resultant ACL damage: http://www.ski-injury.com/specific-injuries/knee

^6^This standardization primarily addresses the ski sports retailers. See www.iso.org

^7^The tibia method is in the process of being removed from all ISO standards, see ISO 8061-2015 and ISO 11088-2018.

^8^Ski types used: *Atomic SL:2.11* (Setup A) 156*cm* (used condition) *Betacarve, Dynastar Legend Pro Rider* (Setup B) 186*cm* (used condition). Contrary ski types and ski binding types were used for reasons of assessing the limits of the testing setup concerning deflection and bending. An *Atomic 614* Race binding (Setup A) has been chosen to represent a standard mid-range priced piste binding as well as *Look Pivot 13* bindings (Setup B). Latter offer a turntable heel piece which is more favored in freeskiing because of its coupling pressure and long elastic travel of the heel piece, which is visible in the pressure curves.

^9^Universal general-purpose strain gauge by *Micro-Measurements*, type *CEA-9.6-187UVA-350*. The full bridges were applied to measure shear forces, which results in temperature compensation, independence of location where the force is applied to the lever and in non-sensitivity to torsion.

^10^For example, it could be detected if the end-effector experienced a pure lateral movement relative to the ski, or if a turning motion was applied.

^11^*BEV - Bundesamt für Eich- und Vermessungswesen*.

^12^Boot-induced anterior drawer is a mechanism caused by a backward fall putting severe stress on the knee structures because of the skier's mass inertia in connection with the stiff boot structure, first characterized in Johnson et al. (1983).

^13^Release motion *3.a* equals *2.c*. *2.c* was plotted again in *3.a* for better clearness and comparison reasons.

^14^such as diagonal release bindings, dual pivot bindings or vertical release bindings.

^15^Movement of tibia and Os femoris.

^16^In a case–control study in Flaine (Laporte et al., 2009), they asserted that 15% lower set binding release values in female skiers than the settings recommended by the ISO 11088 standard would significantly reduce knee injuries.

^17^The are rigorous differences between female and male skiers concerning the knee injury rates (Greenwald and Toelcke, 1997; Donner and Walther, 2007; Ruedl et al., 2009; Ekeland and Hospital, 2016).

^18^Ruedl et al. (2009) assume that the shorter carving skis compared to the longer and straight-shaped traditional skis cause a distribution change of the ACL injury mechanisms from backward twisting fall (29%) to forward twisting fall (51%) among female skiers.

^19^e.g., Kneebinding of Kneebinding, Inc. http://www.kneebinding.com/ The exact effect to knee injury prevention and binding pre-release occurrence seems still unclear (Senner et al., 2014) or Kingpin bindings of Marker GmbH https://www.marker.net/en/products/bindings/touring/kingpin-13/

^20^Ski Binding Boot.

^21^Angular velocities of the six axis vary between 115°/s and 260°/s at 150 kg nominal load capacity.

^22^As the knee itself is a complex structure of a synovial joint and viscoelastic ligaments, of course, the effects of braces and knee proprioception offer a wide field for discussion (Teitz et al., 1987; Requa and Garrick, 1990).
